# Cross-Talk between Malarial Cysteine Proteases and Falstatin: The BC Loop as a Hot-Spot Target

**DOI:** 10.1371/journal.pone.0093008

**Published:** 2014-04-03

**Authors:** Srinivasan Sundararaj, Ajay K. Saxena, Ruby Sharma, Kapil Vashisht, Supriya Sharma, Anup Anvikar, Rajnikant Dixit, Philip J. Rosenthal, Kailash C. Pandey

**Affiliations:** 1 Host–Parasite Interaction Biology Group, National Institute of Malaria Research, Indian Council of Medical Research, Dwarka, New Delhi, India; 2 Structural Biology Laboratory, School of Life Sciences, Jawaharlal Nehru University, New Delhi, India; 3 Department of Medicine, San Francisco General Hospital, University of California San Francisco, San Francisco, California, United States of America; Stanford University, United States of America

## Abstract

Cysteine proteases play a crucial role in the development of the human malaria parasites *Plasmodium falciparum* and *Plasmodium vivax*. Our earlier studies demonstrated that these enzymes are equipped with specific domains for defined functions and further suggested the mechanism of activation of cysteine proteases. The activities of these proteases are regulated by a new class of endogenous inhibitors of cysteine proteases (ICPs). Structural studies of the ICPs of *Trypanosoma cruzi* (chagasin) and *Plasmodium berghei* (PbICP) indicated that three loops (termed BC, DE, and FG) are crucial for binding to target proteases. Falstatin, an ICP of *P. falciparum,* appears to play a crucial role in invasion of erythrocytes and hepatocytes. However, the mechanism of inhibition of cysteine proteases by falstatin has not been established. Our study suggests that falstatin is the first known ICP to function as a multimeric protein. Using site-directed mutagenesis, hemoglobin hydrolysis assays and peptide inhibition studies, we demonstrate that the BC loop, but not the DE or FG loops, inhibits cysteine proteases of *P. falciparum* and *P. vivax* via hydrogen bonds. These results suggest that the BC loop of falstatin acts as a hot-spot target for inhibiting malarial cysteine proteases. This finding suggests new strategies for the development of anti-malarial agents based on protease-inhibitor interactions.

## Introduction

*Plasmodium falciparum* and *P. vivax* are the most predominant human malaria parasites worldwide. According to the latest WHO estimate, 219 million cases of malaria and an estimated 660,000 deaths from malaria occurred in 2010 [1]. Control measures for malaria are seriously hindered by resistance of malaria parasites to many available anti-malarial drugs [2]. Resistance against artemisinins, the most important new class of effective drugs, is also emerging [3]. Therefore, new anti-malarial drugs, particularly acting against new biochemical targets, are needed. Among potential new targets for anti-malarial chemotherapy are *Plasmodium* proteases. Proteases are druggable targets; at present protease inhibitors are licensed and in clinical development to treat multiple diseases, including osteoporosis, diabetes, cancer, hypertension and viral infections.

Among falcipain family cysteine proteases, key enzymes in erythrocytic parasites appear to be falcipain-2 (FP2) and falcipain-3 (FP3), which are major hemoglobinases of *P. falciparum*, and in *P. vivax*, vivapain-2 (VP2), vivapain-3 (VP3) and vivapain-4 (VP4) [4]. Structural and biochemical analysis of falcipains showed that they have specific domains with defined functions. These include the trafficking domain, inhibitory domain, refolding domain and hemoglobin binding domain [5]–[9]. A recent study also clarified the mechanism of auto-activation of falcipains, whereby salt bridges and hydrophobic interactions between the prodomain and the mature domain play crucial roles [10].

Since many cysteine proteases have broad specificity, it is important to regulate their activity. Inhibitors of cysteine proteases (ICPs) of malaria parasites were discovered based on their similarity to the *Trypanosoma cruzi* ICP chagasin [11], [12]. *P. falciparum* expresses the ICP falstatin, which appears to facilitate the invasion of erythrocytes by asexual merozoites by inhibiting host and/or parasite cysteine proteases [13]. Similarly, PbICP, the falstatin homologue in *P. berghei,* appears to facilitate hepatocyte invasion by sporozoites and to block programmed cell death by hepatocytes infected with liver stage parasites [14]. PyICP, the homologue from *P. yoelii*, appears to play a similar role in hepatocytes [14]. Falstatin is a competitive and reversible inhibitor of falcipains [13]. It has previously been shown by structural analysis of chagasin–FP2 and PbICP-FP2 complexes that three ICP loops, known as BC (also named L2), DE (L4) and FG (L6), bind to the target proteases [12], [15]. The C-terminal inhibitory domain of PbICP binds with FP2 in a 1∶1 complex, and the solved structure suggests that PbICP is a member of the I42 class of chagasin-like protease inhibitors.

Structural studies have helped to explain the mechanism of inhibition of falcipains by small molecule inhibitors [16], [17]. For ICPs, the structures of chagasin–FP2 and PbICP-FP2 demonstrated that three loops play important roles in binding to the target proteases. However, it is not known how specifically falstatin interacts with cysteine proteases. In this study we investigated the role in falcipain binding of each loop of falstatin. We found that only the BC loop appears to plays a central role in protease inhibition.

## Materials and Methods

### Ethics Statement

Malaria Parasite Bank of National Institute of Malaria Research, New Delhi, has been approved by ethical committee for *in vitro* culture of *P. falciparum.*

Z- Leu-Arg-AMC (7-amino-4-methylcoumarin) was from Taurus Scientifics. Wild type and mutant peptides were purchased from Biochem Life Sciences, India. Restriction endonucleases, polymerases and T4 ligases were from Fermentas. All other reagents and chemicals were as mentioned in the text.

### Cloning of Wild Type and Mutant Falstatin

Amplification of falstatin fragments was done by PCR using *P. falciparum* cDNA and an earlier described procedure [13]. The amplified DNA fragments were purified by gel extraction, ligated directly into the pGEM-T vector and transformed in *Escherichia coli* JM109 competent cells using a Promega TA cloning kit. The wild type, and mutants (Asn 287, Phe 397) of falstatin were constructed to study the role of BC and FG loops. The wild type, and mutants (Asn 287 to Ala 287, Phe 397 to Ala 397) of falstatin were constructed to study the role of BC (L2) and FG (L6) loops. The signal sequence was deleted, and expressed the wild type and the mutants of falstatin as described earlier [13]. All mutants of falstatin were acquired by overlap extension PCR [18]. Mutant sequences were confirmed by DNA sequencing. Wild type and mutant falstatins were amplified from the falstatin-pGEM-T plasmid, digested with *Bam*HI and *Hin*d III and ligated in a pQE30 expression vector (Qiagen).

### Expression and Purification of Falstatin and its Different Constructs

Various constructs of falstatin were transformed in *E. coli* M15 (pREP4) cells (Qiagen) and expressed with 0.5 mM IPTG at 33°C for 4 hours. The pellets were suspended in 50 mM phosphate buffer pH 8, 500 mM NaCl, 1 mM phenyl methyl sulfonyl fluoride (PMSF), 1 mM benzamidine hydrochloride, 10 mM imidazole, 3 mM β-mercaptoethanol, sonicated with a 20 sec pulse and 1 min gap per cycle for 7 cycles and centrifuged at 12,000 g. The supernatant was then incubated with pre-charged Ni-NTA resin (Qiagen) for 1 hour, washed with 50 mM imidazole and eluted with 100–300 mM imidazole, using EKTA Prime Plus purification system from GE Health Care. The eluted protein was concentrated using a 10 kDa cut-off membrane (Millipore), loaded on Sephacryl S-200 HR gel filtration column pre-equlibrated with 50 mM phosphate buffer pH 8, 150 mM NaCl, 5% glycerol and concentrated to 3 mg/ml. Gel filtration markers (Ferritin, 660 kDa; Aldolase, 440 kDa; ovalbumin, 43 kDa) were from GE Health Care.

### Modeling of Falstatin-FP2, Falstatin-FP3 and Falstatin-VP2 Complexes

The coordinates of crystal structures of mature domains of FP2 (244–284 aa, PDB-1YVB), [9] and FP3 (8–249 aa, PDB-3BWK), [16], [17] were obtained from the NCBI protein database. The Phyre server [19] and Modeler V 9.10 [20] were used to model the structures of VP2 and falstatin.

The structural model of falstatin was obtained using the PHYRE server, which used the chagasin crystal structure (PDB-2OUL), [12] as best input template. We also built the falstatin model with the Modeler V 9.10 program [20] using the PbICP-C crystal structure (PDB-3PNR), [14] as input template. The model was evaluated based on best Z-DOPE score. The initial complexes of falstatin-FP2, falstatin-FP3 and falstatin-VP2 were obtained with the COOT program [21] using chagasin-FP2 (PDB-2OUL), [12] and PbICP-FP2 (PDB-3PNR), [15] as input templates. We used principal protein-protein docking server CLUSPRO [22] to obtain a set of possible complexes. The server yielded the best docking complexes of falstatin-FP2, falstatin-FP3 and falstatin-VP2. Energy minimization was performed using the GROMACS program [23] on all three complexes of falstatin using 200 steps of steepest descent and 500 steps of conjugate gradient algorithm. The protein-protein interactions server [24] was used to identify all interacting residues at complex interface and their solvent accessibility. The free binding energy between both chains of the complex was calculated using the DCOMPLEX program [25].

### Hemoglobin Hydrolysis

To assess the hydrolysis of hemoglobin, 8 μg of hemoglobin was incubated with 300 nM of FP2, FP3, or VP2 with and without 10 μM E-64 or 500 nM falstatin. Wild type and mutant peptides at different concentrations (8–32 μM) were incubated with the above enzymes before incubation with 8 μg of hemoglobin at 37°C for 3 hours. Reaction products were resolved by 15% SDS-PAGE and identified by staining with Coomassie as previously described [26].

To assess hemoglobin hydrolysis by spectrophotometry, proteases as mentioned above were incubated with and without 10 μM E-64 or 500 nM falstatin before adding 15 μg of hemoglobin. The above reactions were incubated in 100 mM sodium acetate, pH 5.5, 8 mM reduced glutathione in 500 μl reaction mixture. Equal amounts of wild type and mutant peptides (16 μM) were incubated with the proteases before adding hemoglobin. For all of these hydrolysis reactions were monitored by measuring absorbance at 410 nm.

To confirm the specificity of the peptide based on the BC loop (LDSV**N^287^**G**N^289^**G**F^291^**VW), 5 μg trypsin (in 100 mM Tris, pH 7.4), 5 μg pepsin (in 100 mM acetate buffer, pH 4.0), or 5 μg plasmepsin 4 (*P. vivax* aspartic protease in 100 mM acetate buffer, pH 4.5), [27] were pre-incubated with and without 16 μM of peptide. After 20 minute incubations, 8 μg of hemoglobin was added and reaction products after 3 hour incubations were analyzed using 15% SDS-PAGE.

### Measurement of Enzyme Activity using a Fluorogenic Substrate

To assess hydrolysis of a fluorogenic peptide, FP3, VP2 and FP2 (30 nM) were incubated with and without 1 μM E-64 and 50 nM falstatin before adding 25 μM Z-Leu-Arg-AMC. The wild and mutant falstatin were incubated in 100 mM sodium acetate, pH 5.5, 8 mM DTT. For peptide mediated inhibition, a wild type or mutant peptides (16 μM) were incubated with 30 nM VP2, FP3 and FP2 for 35 min before adding substrate. Fluorescence (excitation, 355 nm; emission, 460 nm) resulting from hydrolysis of substrate was continuously measured for 15 min at room temperature as described earlier [26], [28].

### Parasite Culture

*P. falciparum* strain 3D7 was cultured in human erythrocytes (procured from the Indian Red Cross Society) using 2% hematocrit in RPMI medium 1640 supplemented with 10% human serum [29]. Synchronization was maintained by treatment with 5% D-sorbitol [30]. Synchronized ring-stage parasites were pre-incubated with wild type peptide (LDSV**N^287^**G**N^289^**G**F^291^**VW) and mutant peptide (LDSV**N^287^**G**A^289^**G**F^291^**VW) based on the BC loop (25.0 μM) or E-64 (10 μM), and after 30 hours of incubation slides were prepared, stained with Giemsa and examined for morphological changes.

### Hemozoin Assay

Synchronized ring-stage parasites (200 μl with 6% parasitemia) were treated with two different concentrations of wild type peptide (25.0 μM and 50.0 μM). After 30 hours slides were prepared to confirm the development of early schizonts.

Schizonts were treated with 800 μl of 2.5% SDS in 0.1 M sodium bicarbonate pH 8.8, and mixed at room temperature for 20 min. The supernatant was removed after centrifugation at 13,000 rpm for 10 min. The pellet was washed twice with 1ml of 2.5% SDS in 0.1 M sodium bicarbonate (pH 8.8), and finally resuspended in 500 μl of 5% SDS and 50 mM NaOH. The quantity of monomeric heme was measured at 405/750 nm as described earlier [31].

## Results

### Similarity between Falstatin and Other ICPs

**Figure 1:**
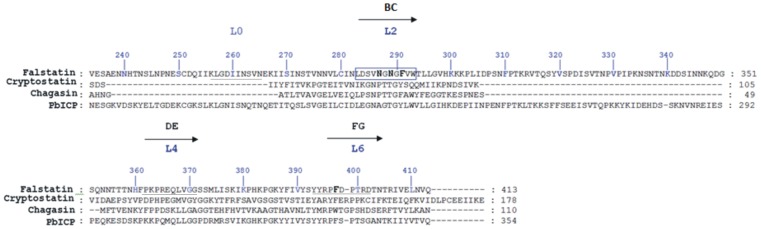
Alignment of falstatin with other ICPs. A multiple sequence alignment was performed with falstatin and ICPs from *Cryptosporidium parvum* (cryptostatin), *Trypanosoma cruzi* (chagasin), and *Plasmodium berghei* (PbICP). This alignment predicted four major loop regions; L0, BC (L2), DE (L4) and FG (L6) in falstatin. The peptides used in this study are underlines, and residues mutated in the described studies are in bold type.

Structural studies of chagasin and PbICP led to the identification of crucial loops in the ICP family of proteins. Falstatin and its plasmodial homologues have molecular masses much higher than those of other ICPs, and so it was unclear if the structural determinants of inhibition by falstatin are similar to those of non plasmodial ICPs [11], [12]. Our first objective was to identify and characterize the determinant of inhibition in falstatin. We aligned the sequences of falstatin and PbICP with those of other ICPs. Falstatin and it plasmodial homologues have long stretches of amino acids that are absent in chagasin and homologues from *Leishmania mexicana* and *Cryptosporidium parvum* [11], [12], [32] (Fig. 1). Overall, the sequence similarity between falstatin and chagasin is ∼20%, and that between falstatin and PbICP is ∼38%. Based on homology with the solved structures of chagasin and PbICP [12], [15], we identified four major loops, labeled as L0, BC (L2), DE (L4) and FG (L6) (Fig. 1).

### Falstatin is a Multimer

**Figure 2:**
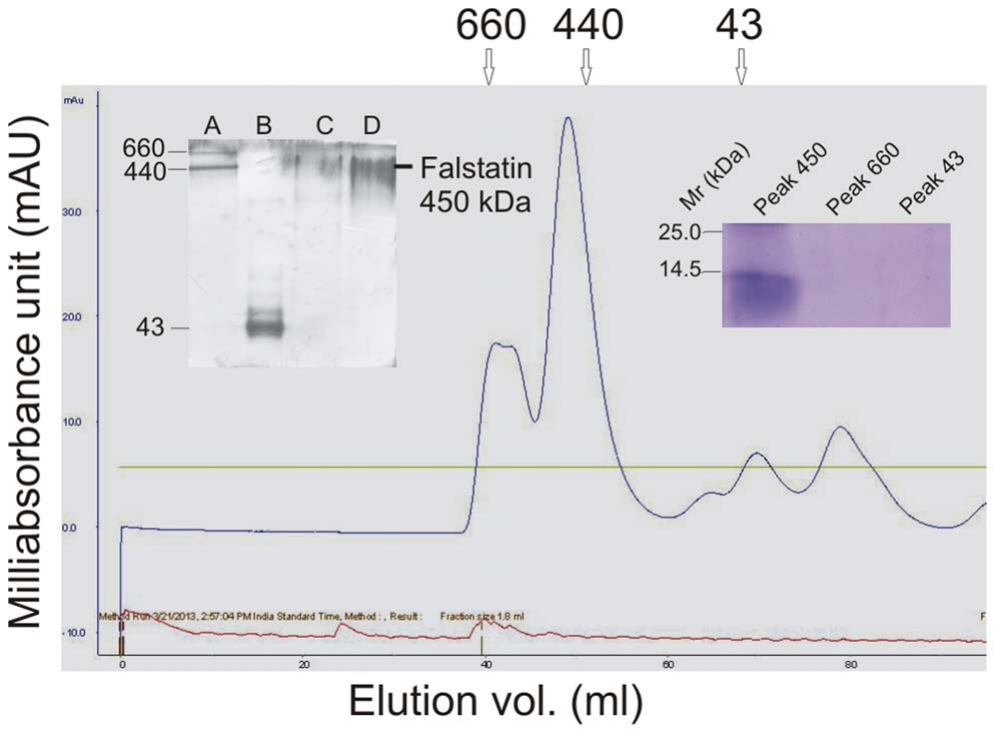
Falstatin is a multimer. Native falstatin was characterized by gel filtration and native PAGE analysis. The elution profile of falstatin and its migration in native PAGE (right inset) predict a size of ∼450 kDa. Lane A: 660 kDa and 440 kDa markers; Lane B: 43 kDa marker; Lane C: 8 μg of active falstatin; Lane D: 12 μg of active falstatin. The functional activities of different peaks were analyzed by hemoglobin hydrolysis observed in 15% SDS-PAGE (left inset). Gel filtration analysis and activity assay of different peaks were repeated more than two times. The blue and red lines indicate the absorbance of proteins and conductance of buffer, respectively.

Under reducing and denaturing conditions the apparent molecular mass of falstatin was estimated to be 45 kDa [13]. Based on elution patterns in gel filtration and analysis with native PAGE (right inset), falstatin appeared to be multimeric, with approximate size of the multimer of 450 kDa (Fig. 2), indicating a complex of 10 units of falstatin. The functional activities of different peaks of gel filtration chromatography suggested that only a 450 kDa peak is active (left inset). This result contrasts with data for PbICP and chagasin, which indicate that these inhibitors function as monomers [12], [15] (Fig. 2).

### The BC Loop Plays a Pivotal Role in Cysteine Protease Inhibition

**Figure 3:**
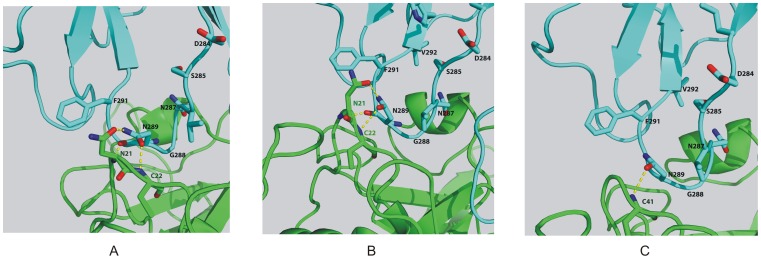
Models depicting the interactions of wild type peptide and VP2 (A), FP3 (B), and FP2 (C). 3D models depict interactions of the BC loop peptides (turquoise) with the active site (green) regions of VP2 (A), FP3 (B) and FP2 via hydrogen bonds and hydrophobic interactions.

The structure of PbICP-FP2 suggests that the C-terminus of PbICP binds to the target protease via its four loops (L0, BC, DE and FG) [15]. To understand the roles of falstatin loops, mutants were designed by changing loop amino acids (Asn^287^ from BC and Phe^397^ from FG) to alanine. These point mutants, wild falstatin (Fig. S1A), and the proteases VP2, FP2 and FP3 were constructed, expressed and purified (Fig. S1B and Fig. S1C). The inhibitory activities of wild and mutant falstatins were compared by measuring their effects on hemoglobin hydrolysis by FP3. Results with the two mutants showed that the mutated residues are not crucial for inhibition of FP3 (Fig. S2). Based on these data, we used molecular modeling of falstatin in complex with VP2, FP3 and FP2 to predict key inhibitory falcipain domains. Our models predicted that the sequence corresponding to the BC loop fits into the active site of VP2 (Fig. 3A), FP3 (Fig. 3B) and FP2 (Fig. 3C).

**Figure 4:**
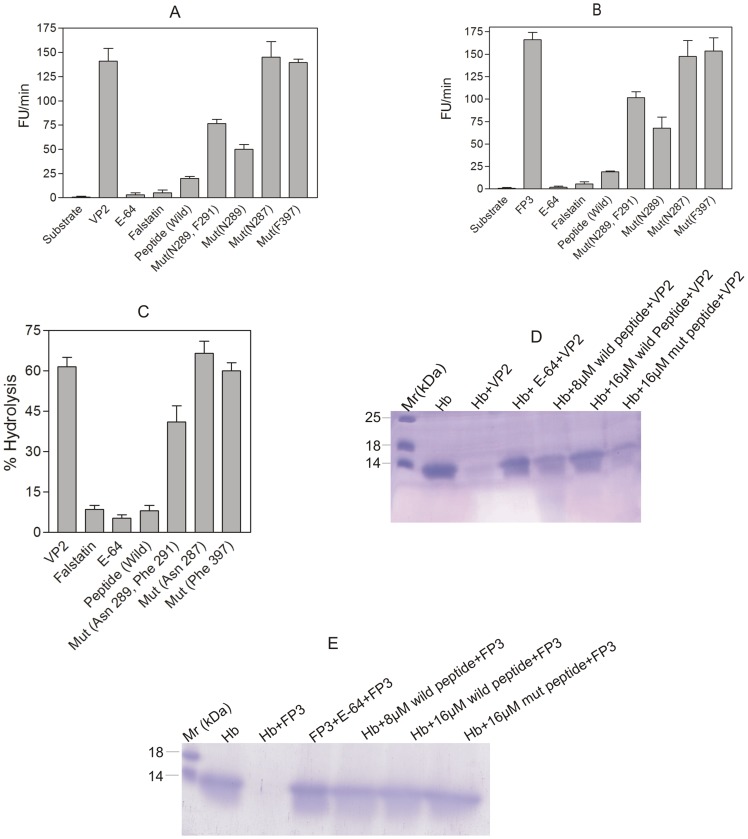
Hydrogen bonds are crucial between the BC loop of falstatin and cysteine protease. Hemoglobin and fluorescence peptide hydrolysis were done in the presence of wild type peptide (LDSV**N^287^**G**N^289^**G**F^291^**VW), mutant peptides (LDSV**N^287^**G**A^289^**G**A^291^**VW and LDSV**N^287^**G**A^289^**G**F^291^**VW), and mutants of falstatin (Asn ^287^, Phe ^397^). The Inhibitory property of wild type peptide and mutant peptides (16 μM) based on a BC loop were assessed by using fluorescence peptide (Z-Leu-Arg-AMC) with VP2 (Fig. 4A) or FP3 (Fig. 4B).The percentage of fluorescence units were measured and calculated for the hydrolysis of the fluorogenic substrate (A, B). Similarly, the Inhibitory property of wild type peptide and mutant peptide were assessed by using spectrophotometer at 410 nm (Fig. 4C). Error bars are shown based on means from two independent assays. Hemoglobin hydrolysis by VP2 (Fig. 4D) and FP3 (Fig. 4E) were also assessed in the presence of wild type (LDSVN^287^GN^289^GF^291^VW) and mutant peptide (LDSVN^287^GA^289^GA^291^VW) under optimal conditions and hydrolysis pattern was observed in 15% SDS-PAGE.

To validate our in silico results, we studied the effects on hydrolysis of a peptidyl substrate and hemoglobin in the presence of a wild type peptide (LDSV**N^287^**G**N^289^**G**F^291^**VW) based on the BC loop of falstatin. The peptide caused a >75% decrease in hydrolysis of the fluorogenic substrate, Z-Leu-Arg-AMC (Fig. 4A, 4B). The peptide also inhibited the hydrolysis of the natural substrate hemoglobin, measured spectrophotometrically, by VP2 (Fig. 4C) or, measured by SDS-PAGE, by VP2 or FP3 (Fig. 4D and 4E).

**Figure 5:**
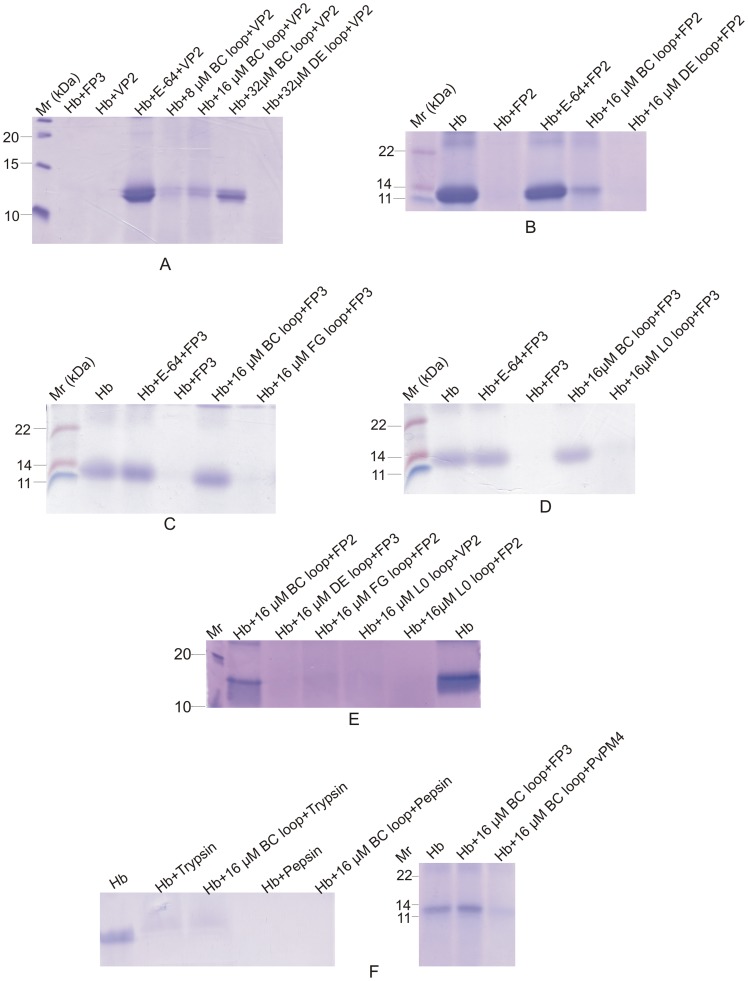
Only the BC loop appears to plays a central role in protease inhibition. Peptides based on the DE, FG and L0 loops of falstatin also incubated with VP2 (Fig. 5A) or FP2 (Fig. 5B) or FP3 (Fig. 5C, Fig. 5D add E), and hemoglobin hydrolysis patterns were observed in 15% SDS-PAGE. As a control the wild type peptide was pre-incubated with trypsin, pepsin, and plasmepsin- 4 of *P.vivax* (PvPM4) under optimal conditions and hydrolysis pattern was observed in 15% SDS-PAGE (5F). The hydrolysis of hemoglobin and fluorogenic substrate was done with two independent batches of each enzyme.

In contrast, peptides based on the DE, FG and L0 loops of falstatin did not inhibit hemoglobin hydrolysis by VP2 (Fig. 5A) or FP2 (Fig. 5B) or FP3 (Fig. 5C and Fig. 5D and 5E). Although, there was a difference in inhibitory potency between the wild type peptide and falstatin, but both were efficient inhibitors of malarial cysteine proteases. The difference of inhibition between the wild type and the mutant peptide in two different assays (Fig. 4B and 4E) was due to different level of sensitivity. The hydrolysis of hemoglobin, by proteases of other classes, the serine protease trypsin and the aspartic proteases pepsin and *P.vivax* plasmepsin-4 (PvPM4), [27] was unaffected by the peptide (Fig. 5F). Thus, the BC loop peptide mimicked wild falstatin to block hemoglobin hydrolysis by plasmodial cysteine proteases.

### Hydrogen Bonds are Crucial for the Inhibitory Activity of Falstatin

The structural analysis of PbICP and chagasin suggested the importance of the BC loop (LDSV**N^287^**G**N^289^**G**F^291^**VW) in inhibition of cysteine proteases [12], [15]. To understand the interactions that govern the inhibitory activity of falstatin, homology modeling energy minimization was carried out. These studies suggested that Asn^289^ of falstatin, which lies in the BC loop, forms hydrogen bonds with Asn^21^ and Cys^22^ of VP2, and with Cys^41^ of FP3 (Fig. 3) and also hydrophobic shielding between Phe^291^ of the inhibitor and the surrounding active site regions of VP2 and FP3. To analyze the role of predicted hydrogen bonding and hydrophobic interactions in the BC loop of falstatin, two mutant peptides (LDSVN^287^G**A^289^**G**A^291^**VW and LDSVN^287^G**A^289^**G**F^291^**VW) were synthesized and did hemoglobin hydrolysis assay. Comparison of double (N289A F291A) and single mutations (N289A) with the wild type peptide, it was suggested that only asparagine (N289) residue was required for efficient inhibition (Fig. 4 AB). Since no additive effect of double mutation was seen, our result suggested that only hydrogen bonds between the BC loop of falstatin and malarial proteases (VP2, FP3) are required for efficient inhibition (Fig. 4 AB). Further, hemoglobin hydrolysis using spectroscopy and SDS-PAGE analysis suggested that the BC loop mutant peptides were slow inhibitor of VP2 and FP3 compare to wild type peptide (Fig. 4 A–E).

### Effect of the Falstatin BC Loop on *P. falciparum* Trophozoites

**Figure 6:**
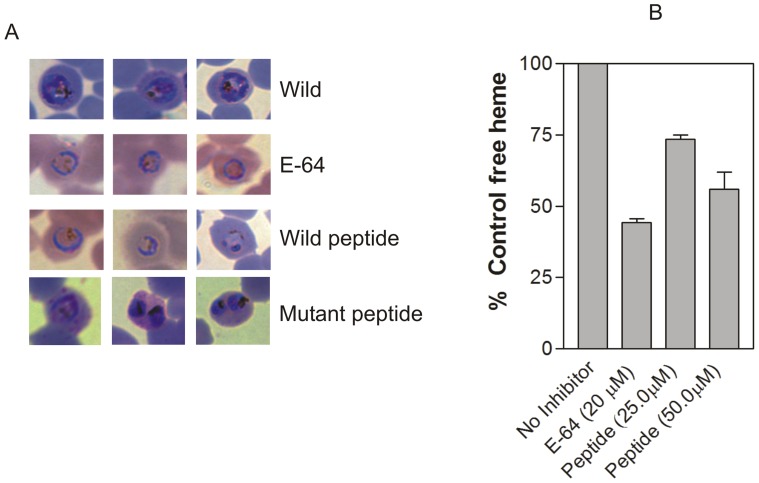
Effect of BC peptide on cultured *P. falciparum*. A Morphology of parasites treated with wild type and mutant BC peptides. Synchronized ring-stage parasites were incubated with the wild type (LDSVN^287^G**N^289^**G**F^291^**VW) and the mutated peptide (LDSVN^287^G**A^289^**G**F^291^**VW) (25.0 μM) or E-64 (10 μM), and after incubation parasites were stained with Giemsa. Morphology of parasites was analyzed with two independent cultures of *P. falciparum*. B. **Impact of wild type BC peptide on free heme formation.** Synchronized ring-stage parasites were incubated without inhibitor or with the peptide or E-64 at the indicated concentrations, and after 30 hours free heme was quantified by spectroscopy. Percentages of control free heme are shown based on means from two independent assays.

The entry of fluorescent peptides (mol. wt. range from 653 to 3200 Da) into parasitized red blood cells has been previously investigated [33] and shown that peptides up to 2365 Da can be transported into the parasite. Similarly to test the effects of the wild type BC loop peptide (LDSVN^287^G**N^289^**G**F^291^**VW) in intact parasites, *P. falciparum* rings were incubated with the wild type and mutant peptides (LDSVN^287^G**A^289^**G**F^291^**VW), and after 30 hours of incubation, slides were prepared and parasites were examined (Fig. 6A). There was a clear morphological change in wild type peptide-treated parasites, with the appearance of a swollen, dark-staining food vacuole, as seen after incubation with the cysteine protease inhibitor, E-64 (Fig. 6A). To rule out the nonspecific toxicity effect of the wild type BC loop peptide, equal concentration of a mutant peptide (LDSVN^287^G**A^289^**G**F^291^**VW) was added in the culture as a control (Fig. 6A). This experiment suggested that the wild type BC loop peptide specifically inhibited the parasite cysteine proteases. We also estimated parasite production of free heme, a marker of hemoglobin hydrolysis [31]. We saw a dose-dependent reduction in free heme formation in peptide treated compared to untreated parasites (Fig. 6B).

## Discussion

We previously showed that falstatin, an endogenous cysteine protease inhibitor of *P. falciparum*, appears to play a key role in the invasion of erythrocytes by merozoites of *P. falciparum*. Further, the falstatin homolog PbICP appears to play a key role in hepatocyte invasion by sporozoites [14]. Our present study focuses on understanding the interactions responsible for falstatin inhibitory function. Multiple sequence analysis identified three falstatin loops which shared moderate similarity (<40%) with the related ICPs chagasin and PbICP, and computational analysis suggested key hydrogen bonds and hydrophobic interactions between falstatin and falcipains. Specifically, a peptide representing the falstatin BC loop, but not peptides representing the DE or FG loops, inhibited FP2, FP3 and VP2, demonstrating the key role of this loop in mediating falstatin function. Parasite treated with peptide showed distinct food vacuole abnormalities with undegraded hemoglobin, further hemozoin assay correlated the dose dependent inhibition of malarial cysteine proteases, which was further evidenced by the reduction in hemoglobin degradation. Mutation of residues predicted to mediate inhibitor-enzyme interactions ablated the inhibitory function of the peptide. Taken together, these results demonstrate the key role of the falstatin BC loop in mediating endogenous inhibition of cysteine proteases by *P. falciparum*.

The complex of chagasin-FP2 and PbICP-FP2 demonstrated the importance of BC, DE and FG loops, which bind to the target proteases. However, using peptides based on these loops of falstatin suggest that only a BC loop inhibit the malarial cysteine proteases of *P. falciparum* and *P. vivax*. It was further reported in *Leishmania mexicana* ICP, DE loop peptide also have no direct role in inhibition [34]. It may be possible that DE and FG loops are not require for direct inhibition but may play important role in stability of falstatin-cysteine protease complex. Nonetheless, the interaction between the binding loop and the active site are not conserved in both the endogenous inhibitors. In case of PbICP, Gly^231^ of the inhibitor and Trp^206^ of FP2 form a hydrogen bond [15], while in case of falstatin, Asn^289^ of the inhibitor forms three hydrogen bonds with Asn^21^ and Cys^22^ of the active site of VP2, and one hydrogen bond with Cys^41^ of the active site of FP3. Inhibition of a wide range of cysteine proteases by macromolecule inhibitors like falstatin, PbICP and chagasin, suggest that the BC loop can adopt more than one alternative conformation for optimal and efficient inhibition. But neither falstatin nor PbICP is able to inhibit cathepsin B, because of a loop region that occludes substrate from binding to the primed subsites of the active site cleft. The steric repulsion between the two loops (L3 and L4) of PbICP and the occluding loop of cathepsin B has been reported which inhibit the interaction between protease and inhibitor [15].

Unlike all other known monomeric endogenous inhibitors, falstatin is multimeric forms and thus may favor some additional level of interactions. The close homolog in *P. Berghei*, PbICP is active in monomeric state having binding site in a single cavity, but multimerization of falstatin may cause allosteric modulation of inhibitory potency. The different structural state of falstatin may be reason to inhibit distant related cysteine proteases, human caspase-3, caspase-8 and calpain-1, unlike a closely related homologue, PbICP. As discussed earlier unlike PbICP, falstatin blocks proteolytic activity of caspases and calpain-1 in micro molar ranges [13]. The possible reasons may be besides it multimeric forms, falstatin structure is closer to serpins family and seems to have more flexible loops compare to PbICP. Since falstatin appear as multimeric forms as compare to PbICP, and thus it may also interact with caspases and calpains differently. Inhibition of caspases and calpain-1 by falstatin may be helpful in programmed cell death and to protect the exposed merozoite from host proteases like caspases and calpains. Therefore, it will be very interesting to solve the complex structure of falstatin with cysteine protease. The structural study of falstatin will open new dimension in the field of inhibition of proteases by large protein.

Since a wild type peptide based on falstatin BC loop inhibited the protease activity of recombinant enzymes. We also tested the activity of the peptides in parasite infected RBC. The post incubation of the parasite treated with a peptide showed distinct food vacuole abnormalities with undegraded hemoglobin, suggested that activities of native cysteine proteases can be blocked by a BC loop peptide. The incubation of a mutant peptide did not show similar food vacuole abnormalities suggested that phenotypic effect was not due to nonspecific toxicity. To further evaluate the effects of a wild type peptide on an *in vitro* culture of *P. falciparum*, free heme measurement was done. Earlier reports stated that free heme was generated because of hemoglobin hydrolysis by proteases, and free heme produced should be directly proportional to the extent of hemoglobin degradation. A dose-dependent reduction in free heme formation in peptide treated compared to untreated parasites further confirmed that a peptide based on BC loop inhibited the activities of cysteine proteases in *P. falciparum* culture.

Designing inhibitors based on protein–protein interactions (PPIs) is a new approach especially in malaria. A recent study explained the inhibition of calpain by mimicking a natural protein-protein interaction between calpain and its endogenous inhibitor, calpastatin [35]. Furthermore, researchers aimed to block the interactions of two monomers, and prevent it forming the active dimer interface, and developed the first small molecule inhibitor of a herpes virus protease that blocked PPIs [36]. Since falstatin is released upon schizont rupture, and antibodies that inhibited falstatin action specifically blocked merozoite invasion of erythrocytes, further suggesting that the inhibitor functions to prevent inappropriate activity by parasite and/or host cysteine proteases, and thereby facilitates erythrocyte invasion. Therefore, PPIs between cysteine proteases and falstatin may be targeted in future. Designing inhibitors based on PPIs may be less prone to drug resistant mutation because drug resistance mutation seems less likely to be tolerated by a complex protein-protein interface.

Although our models predicted that the hydrogen bonds and hydrophobic interactions between the BC loop and active sites of malarial cysteine proteases are crucial for strong inhibitory potency of falstatin. But mutagenesis study further confirmed that only hydrogen bonds between the BC loop of falstatin and malarial proteases are required for efficient inhibition. Our results identify the first biochemical evidence of the crucial interactions that is governing the functional property of falstatin, and indicates that falstatin is the first known endogenous inhibitor function as multimeric forms. The Inhibition of enzymes by BC loop provides insights into protein-protein interactions during proteases-inhibitor cross talk. This information can be taken as an interesting starting point in exploring the mechanism of falstatin function and may be helpful in drug development against malarial cysteine proteases.

## Conclusion

The BC loop of falstatin is a target for mediating inhibition of cysteine proteases of malaria parasites via hydrogen bonds.

## Supporting Information

Figure S1 Expression, purification and refolding of VP2, FP3 and falstatin constructs. A; The wild falstatin and its point mutations (Asn^287^ and Phe^397^) were constructs, expressed and purified, SDS-PAGE showing different elution of VP2 (B) and FP3 (C), purified by Ni-NTA chromatography using imidazole gradient.
(TIF)

Figure S2 Effect of falstatin mutants on hemoglobin hydrolysis. FP3 was incubated with falstatin mutants (Asn^287^ and Phe^397^) or E-64 and hemoglobin in 100 mM acetate buffer (pH 5.5), and hemoglobin hydrolysis was assessed with 15% SDS-PAGE.
(TIF)
