# Supplementary figures and images for: Cross-Talk between Malarial Cysteine Proteases and Falstatin: The BC Loop as a Hot-Spot Target
Source: PLoS One. 2014 Apr 3;9(4):e93008. doi: 10.1371/journal.pone.0093008 (PMC3974720; doi:10.1371/journal.pone.0093008)

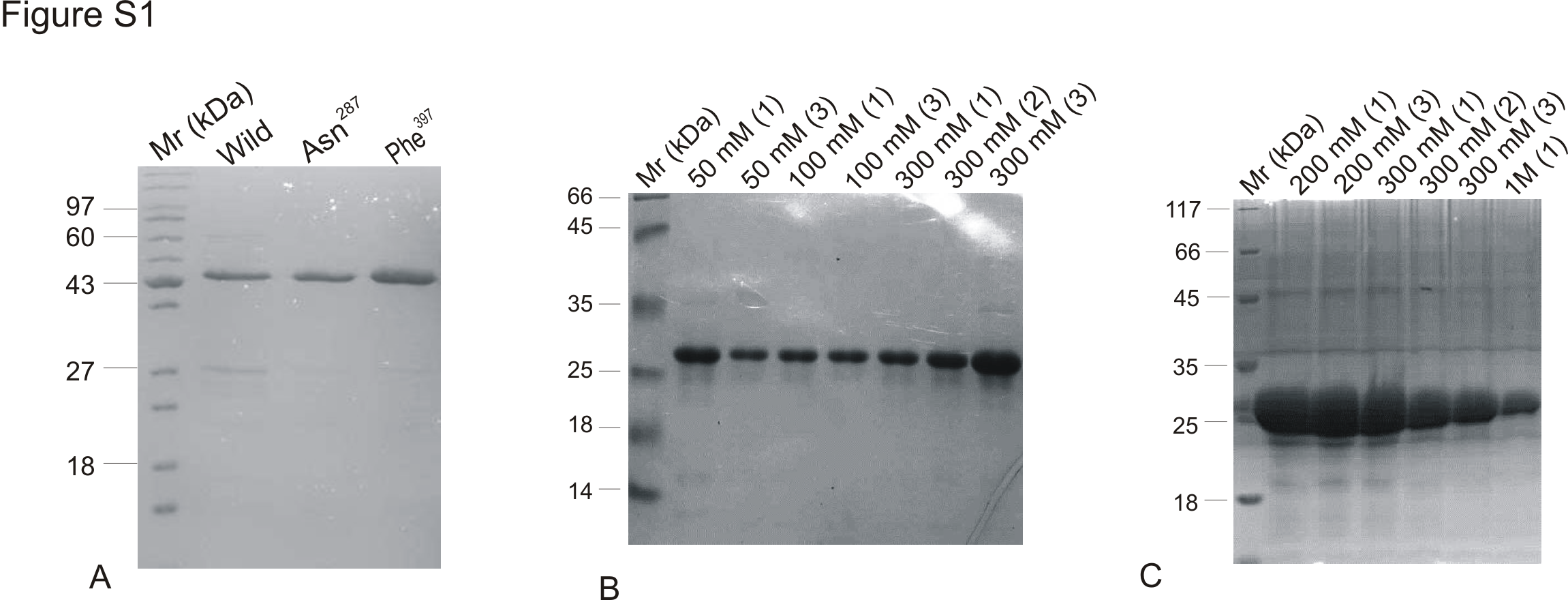

Supplement: Figure S1 — Expression, purification and refolding of VP2, FP3 and falstatin constructs. A; The wild falstatin and its point mutations (Asn287 and Phe397) were constructs, expressed and purified, SDS-PAGE showing different elution of VP2 (B) and FP3 (C), purified by Ni-NTA chromatography using imidazole gradient. (TIF) [file pone.0093008.s001.tif]

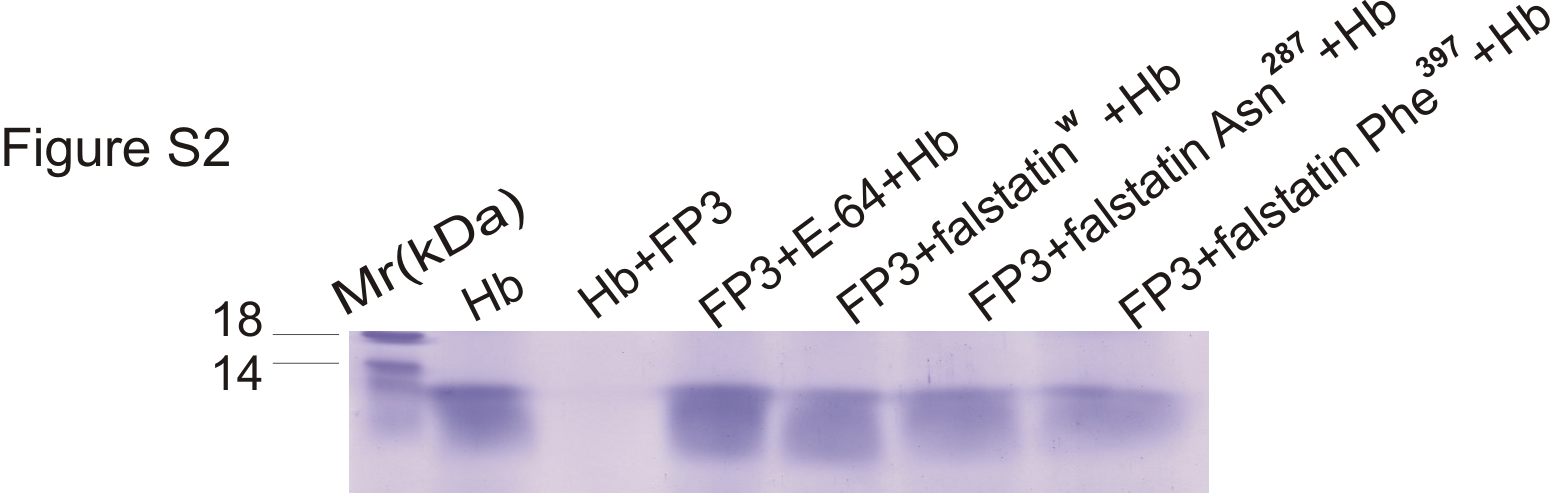

Supplement: Figure S2 — Effect of falstatin mutants on hemoglobin hydrolysis. FP3 was incubated with falstatin mutants (Asn287 and Phe397) or E-64 and hemoglobin in 100 mM acetate buffer (pH 5.5), and hemoglobin hydrolysis was assessed with 15% SDS-PAGE. (TIF) [file pone.0093008.s002.tif]
